# Distribution Patterns of Wood-Decay Macrofungi (Agaricomycetes) in Floodplain Forest Islands of the Eastern Amazon

**DOI:** 10.3390/jof11040288

**Published:** 2025-04-07

**Authors:** Vitória Pinto Farias, Maria do Perpétuo Socorro Progene Vilhena, Antonio Walison Gondim-Vieira, Richard Bruno Mendes-Freire, Renan Domingues Pacheco, Braian Saimon Frota da Silva, Adriene Mayra da Silva Soares

**Affiliations:** 1 Programa de Pós-Graduação em Ciências Biológicas, Universidade Federal Rural da Amazônia, Avenida Presidente Tancredo Neves, 2501, Terra Firme, Belém 66077-830, PA, Brazil; vitoriapintofarias@gmail.com (V.P.F.); sprogene@ufra.edu.br (M.d.P.S.P.V.); gondimwalison@gmail.com (A.W.G.-V.); richard.b.m.freire@gmail.com (R.B.M.-F.); 2 Coordenação de Botânica, Museu Paraense Emílio Goeldi, Avenida Perimetral, 1901, Terra Firme, Belém 66077-830, PA, Brazil; 3 Programa de Pós-Graduação em Ecologia, Universidade Federal do Pará, Rua Augusto Corrêa, Guamá, Belém 66075-110, PA, Brazil; renandomingues013@gmail.com; 4 Instituto de Ciências Exatas e Naturais, Federal do Pará, Rua Augusto Corrêa, 01, Guamá, Belém 66075-110, PA, Brazil; braiansaimon@yahoo.com.br; 5 Laboratório de Botânica e Micologia, Universidade Federal Rural da Amazônia, Rodovia PA-451, Km-03, Açaizal, Tomé-Açu 68680-000, PA, Brazil

**Keywords:** amazonian ecosystems, conservation, seasonal dynamics, wood-decay fungal communities

## Abstract

Macrofungi are key decomposers of organic matter and play an active role in biogeochemical cycles, thereby contributing to carbon sequestration in forest ecosystems. Floodplain forests (várzeas) are characterized by the dynamics of rising and receding waters, which are rich in suspended material and influence species variation and adaptation. The knowledge about the distribution of macrofungi in várzea environments in the Brazilian Amazon is limited. This study aims to evaluate the diversity and composition of macrofungi on three várzea forest islands, while also examining differences in species richness and abundance between seasonal periods. A total of 88 macrofungal species that belong to the phylum Basidiomycota were identified. The findings revealed significant variations in species composition, yet no notable differences in species richness or abundance were observed between the seasonal periods. The environmental conditions and resources available to macrofungi appear to be consistent among the islands, which leads to a balanced diversity. However, additional research is essential to uncover the true distribution patterns of macrofungi in the várzeas of the Brazilian Amazon, an area under significant threat to its biodiversity.

## 1. Introduction

The Amazon is represented by different ecosystems that are characterized according to the type of predominant vegetation and the influence of seasonal regimes of rivers. In addition to ombrophilous forests, igapó and floodplain forests show a peculiarity: they are constantly fertilized by the rising and falling waters of the rivers, which determines the variation and adaptation of the species present [1]. Just as the level of the rivers interferes with the dynamics of organisms, the composition of their waters also plays an important role in the contribution of organic matter. While blackwater rivers, common in the igapó areas, have low levels of sediment and nutrients, whitewater rivers, rich in suspended material, fertilize the floodplain areas, which constitute the second largest plant formation in the Amazon [2,3,4].

Floodplain forests are ecosystems that store a large amount of carbon in the soil and actively participate in river dynamics and regulating biogeochemical cycles, thus making them a unique habitat for adapted species of animals, plants, and fungi [4]. Some species are strongly adapted and have a preference for certain flooded habitats and, in view of this, their unique ecological functions can be sensitive to climate and environmental changes [5]. Studies show that some species either tolerate or are resistant to disturbances such as severe floods or droughts, while others may be impacted or even lost [6,7].

In addition, due to the seasonal flow of rich nutrients from the sediments, the substrates of floodplain areas are highly fertile and become widely used by local communities in the Amazon in the cultivation, mainly of açaí palms (*Euterpe oleraceae* Mart.), cassava (*Manihot esculenta* Crantz), and cocoa (*Theobroma cacao* L.) [8]. Due to this dynamic ecosystem, these floodplain forests are subject to anthropogenic impacts caused by both agriculture and logging, which can cause losses in floodplain biodiversity [9]. Therefore, the floodplain is a naturally sensitive region, both in relation to temperature variations and rainfall levels and to human-induced impacts [10,11].

Macrofungi are members of the phyla Ascomycota and Basidiomycota and are part of the community of organisms present in floodplain ecosystems. The vast majority of these macrofungi belong to the class Agaricomycetes (Basidiomycota) and are known as mushrooms and wood ears (due to the characteristic shape of their basidiomes). The species can be parasitic, symbiotic, and/or saprophytic and are among nature’s main decomposers of organic matter [12]. Among the parasitic species, *Moniliophthora perniciosa* (Stahel) Aime and Phillips-Mora has a wide spectrum of hosts, including plants of economic importance such as cocoa, which causes serious economic and social impacts in areas that include floodplain plantations in the Amazon [13]. On the other hand, ectomycorrhizas establish symbiotic associations with plant roots and are essential for growth, protection, and tolerance in contaminated soils [14,15].

However, the vast majority of species act as saprophytes and actively participate in the cycling of elements. The conversion of the substrate and consequent release of carbon and nitrogen to the environment occurs in different ways, depending on the enzymatic arsenal of the fungus. In this way, organisms can be classified into different categories; for example, those that cause white rot are able to degrade lignin, cellulose, and hemicellulose, the three main polymers of wood. The species that cause brown rot, on the other hand, degrade only cellulose and hemicellulose, and those that cause soft rot secrete cellulase enzymes that cause a characteristic pattern of decomposition [16].

In addition to playing a fundamental ecological role in nature, research has shown that macrofungi, as well as plants, also present a wide variety of bioactive compounds with some type of action and/or health benefit. In fact, the demand for macrofungi has been increasing globally among consumers due to their outstanding taste and nutritional capacity as a result of the presence of polysaccharides, vitamins, flavones, and unsaturated fatty acids. In turn, the therapeutic capacities are associated with the immunomodulatory, antimicrobial, and antioxidant abilities of these bioactive substances, which have an important effect in the fight against cancer, diabetes, obesity, and hypertension, for example [17]. Depending on how they act in the ecosystem, biotic and abiotic factors exert selective pressures that interfere with the frequency and distribution of species. As important as temperature, seasonal variations in river levels and the contribution of organic matter, and the construction of housing and land use by riverine populations (mainly in monocultures) demand even more from the adaptive processes of fungi in the floodplain environment. Most studies in floodplain forests are related to floristic inventories, diversity, and composition [18,19,20,21], as well as effects of the flood pulse and soil organic matter on vegetation [22,23]. However, knowledge about macrofungi in these areas is still scarce. Since knowledge on the dynamics of the macrofungi species from the class Agaricomycetes in these flooded areas is scarce, this study aims to verify the diversity and composition of macrofungi in three floodplain forest islands, as well as the differences in richness and abundance between seasonal periods.

## 2. Materials and Methods

### 2.1. Study Area

This study was conducted in the state of Pará, in the municipality of Mocajuba, located in the mesoregion of northeastern Pará and in the microregion of Cametá (02°35′31″ S, 49°28′60″ W). The predominant ecosystems are those of terra-firme—with the islands presenting a dense alluvial ombrophilous forest type vegetation—and the floodplain areas, managed and exploited by the riverine population in the form of monocultures and for agroforestry based on cocoa plantations [24,25].

**Figure 1:**
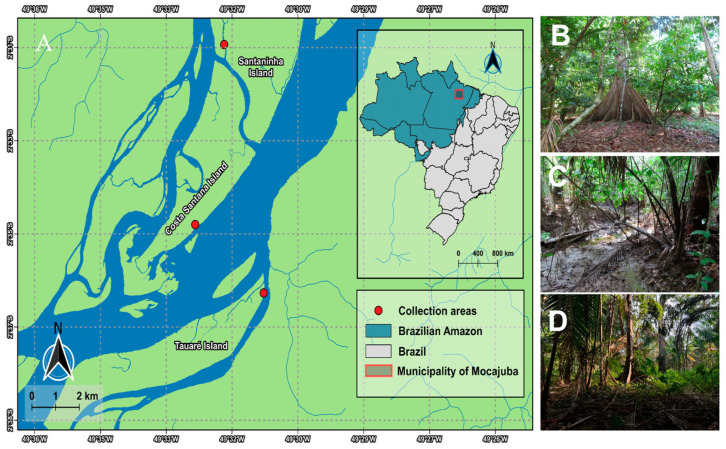
(**A**) Map of the studied region of the municipality of Mocajuba. (**B**) Costa Santana Island; (**C**) Santaninha Island; (**D**) Tauré Island.

The patterns related to humidity, rainfall, and temperature in the municipality change according to the months of the year. The average temperatures range between 25 °C and 34 °C; when the temperature indices begin to increase in July, humidity and precipitation decrease, as do the water levels on the islands, determining the arrival of the dry season [26] and making it possible to access the trails on the islands (Figure 1). Three islands were selected as study areas: Tauaré, Santaninha, and Costa Santana. The selected areas are marked by native cocoa plantations and the predominance of vegetation is mainly characterized by *Hevea brasiliensis* (Willd. ex A. Juss.) Müll.Arg., *Euterpe oleracea*, *Virola surinamensis* (Rol. ex Rottb.) Warb., *Pterocarpus santalinoides* L’Hér. ex DC., and *Carapa guianensis* Aubl. (Table 1).

### 2.2. Collection, Processing, and Identification

The collections were carried out in June and November of 2022 and 2023. The seasons chosen for the field trips mark the end of the rainy and dry periods, respectively, when the levels related to precipitation, humidity, and temperature vary significantly. In the selected floodplain areas, plots of two hectares were demarcated with the help of GPS, which could be covered in a period of two hours. The stages of collection, preservation, and herborization were based on the methodology of Fidalgo and Bononi [27] and Neves et al. [28]. Photographic records of the specimens were made, as well as the description of the substrate and notes on the main characteristics of the basidiomes (size, color, and consistency). The specimens were dehydrated in an electric oven.

Microscopic analyses were performed from freehand sections of the hymenial surface, the context, and the abhymenial surface with the help of steel blades. The fragments were placed on a slide with an aqueous solution of 3–5% potassium hydroxide and 1% phloxin. For cyanophilic observations, Amann’s Blue was used, which stains the walls of hyphae, basidiospores, and cystidia blue. Melzer’s reagent was used in the observation of amyloid (grayish, bluish, or purplish) or dextrinoid (reddish-brown) reactions of the walls of the basidiospores, hyphae, and other microstructures. The observation of the hyphalic system, shape, and size of fertile and sterile followed Teixeira [29]. The slides were observed under an optical microscope for the analysis and measurement of microscopic structures. For the identification, the works of Ryvarden [30,31,32,33,34], Zabin et al. [35], and Gomes-Silva et al. [36] and the specific literature for each taxonomic group were used.

### 2.3. Statistical Analysis

Species diversity was verified by means of relative frequency measurements between the different floodplain areas selected in this study, in addition to the estimation of richness indices. For the diversity analysis, the Shannon–Wiener index (represented by *H*’) was chosen, which was calculated based on the number of individuals of each species and the total number of specimens sampled. The higher the Shannon–Wiener index, the greater the diversity of the sample [37]. The relative frequency of the species was based on the number of specimens and determined by the following equation, F = n/N × 100, where n is the number of specimens of a species and N is the total number of individuals found [38]. The following frequency classes were considered regarding the occurrence of the species: 0.5 < F ≤ 1.5% = rare; 1.5 < F ≤ 5% = occasional; 5 < F ≤ 10% = frequent; F > 10% = abundant; while relative abundance used the equation [number of individuals of a species (N_specimens_)/total number of individuals on the island (N_total_) times 100] [39].

$$Relative\;Abundance\left(\%\right)=\frac{N_{\left\{specimens\right\}}}{N_{\left\{total\right\}}}\times 100$$

Analyses involving species richness and composition between islands were based on the Jaccard index, which measures similarity between finite sample sets through the relationship between the intersection and the size of the union of these same sets. The hypothesis of heterogeneity between the three islands was investigated with the aid of PERMDISP (permutational analysis of multivariate dispersions) and PERMANOVA (permutational multivariate analysis of variance) tests. In addition, the species accumulation curve demonstrated the relationship between sampling effort and observed richness, while the bootstrap method was used to calculate the estimates of richness accompanied by a confidence interval, thus increasing the reliability of the results. All the statistical analyses were performed in R 3.5.2 [40].

To verify the influence of seasonality (dry and rainy periods) on the abundance of macrofungi (n = 88) on the three islands, we used the analysis of variance (ANOVA). This method was selected for its effectiveness in comparing means between groups and identifying possible significant differences related to seasonal conditions. To complement the analysis, the Tukey post hoc and Fisher tests were applied, which allowed for us to detail the differences between the groups, providing different levels of confidence in the results. The box plot graphs were generated using MINITAB software version 14.13 (OSB Software, Bela Vista, SP, Brazil).

## 3. Results

### 3.1. Macrofungal Diversity, Richness and Composition Among the Islands

**Figure 2:**
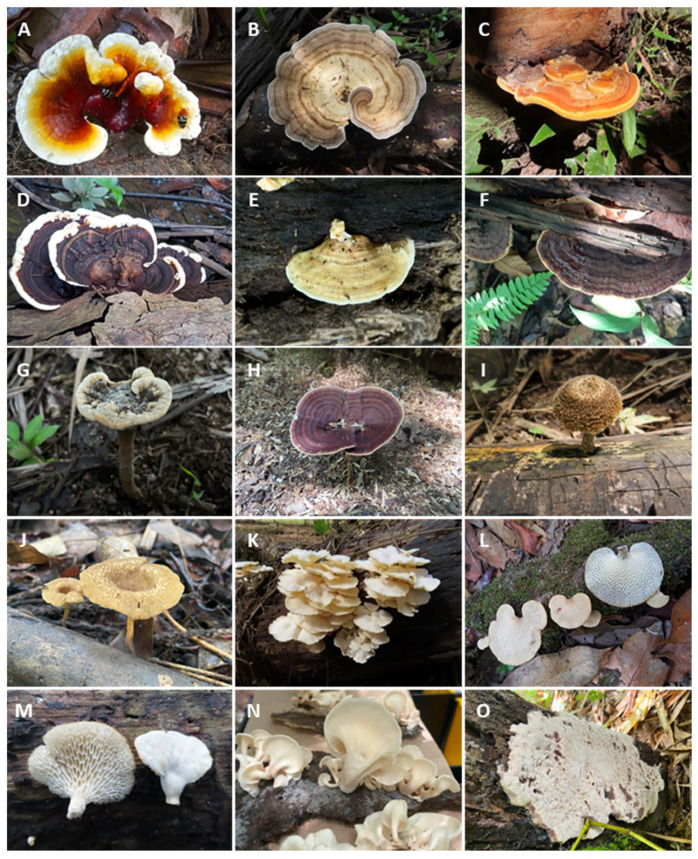
Macrofungi from the flood forests studied in Brazilian Amazon: (**A**) *Ganoderma stipitatum*, (**B**) *Trametes elegans*, (**C**) *Rigidoporus ulmarium*, (**D**) *Earliella scabrosa*, (**E**) *Flaviporus liebmannii*, (**F**) *Cerrena caperata*, (**G**) *Foraminispora rugosa*, (**H**) *Amauroderma exile*, (**I**) *Lentinus swartzii*, (**J**) *Lentinus* sp., (**K**) *Lentinus concavus*, (**L**) *Favolus trigonus*, (**M**) *Favolus tenuiculus*, (**N**) *Pleurotus djamor*, (**O**) *Truncospora tephropora*.

In all, 223 specimens of fungi were identified, representing 88 species belonging to 18 families from the class Agaricomycetes, of the phylum Basidiomycota (Figure 2; Table 2). The order Polyporales presented eleven families, while Agaricales and Hymenochaetales presented three families each, and Russulalles only one. Of the 17 families of Agaricomycetes identified in morphological analyses, Polyporaceae had the highest number of species collected in the floodplain islands (38), followed by Meripilaceae and Hymenochaetaceae with 12 and 8, respectively. Regarding the genera, *Rigidoporus*, *Lentinus*, and *Trametes* were the most diverse, with 12, 10, and 9 species, respectively, while 24 genera presented only a single species. When the absolute number of species and specimens was compared among the islands, Costa Santana presented the highest number of identified species (50) and the highest number of specimens collected (90). Santaninha island occupied the second place, with 37 species and 66 specimens, which was closely followed by Tauaré, with 36 species and 67 specimens.

*Trametes elegans* (Spreng.) Fr. (19 specimens) and *Lentinus berteroi* (Fr.) Fr. *Rigidoporus lineatus* (Pers.) *Ryvarden* (12 specimens) were considered to be frequent, while *Earliella scabrosa* (Pers.) Gilb. and Ryvarden was noted as being abundant (23 specimens). Each island analyzed in isolation showed at least one of these macrofungi in its list of abundant species.

*Earliella scabrosa* (12 specimens) was the most abundant species, while *Trametes elegans* (9 specimens) and *Trametes maxima* (Mont. A. David and Rajchenb (5 specimens) were the most frequent species in Costa Santana according to frequency classes. *Rigidoporus lineatus*, unlike on the other two islands, was one of the most representative species in Santaninha (with 8 specimens), with *Trametes elegans* (9) and *Polyporus guianensis* Mont. (4 specimens) occupying second place. As in Costa Santana, the most abundant species in Tauaré was *Earliella scabrosa* (10), followed by *Podoscypha aculeata* (Berk. and M. A. Curtis) Boidin (7) and *Lentinus berteroi* (7) (Table 2).

Analysis of the totality of the data provided a Shannon–Wiener index equivalent to 3.91 for the three islands. As evidenced by the absolute number of species and specimens collected, Costa Santana presented the greatest diversity among the three islands (3.54) (indicated by the respective Shannon–Wiener index), followed by Santaninha (3.30) and Tauaré (2.99).

**Figure 3:**
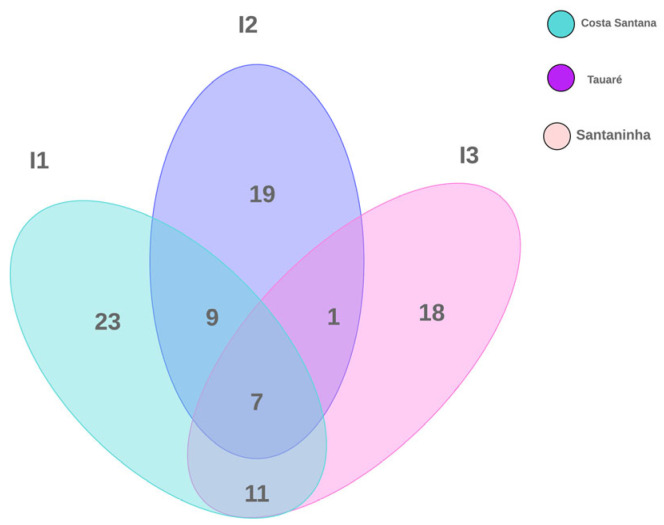
Venn diagram showing the total number of macrofungal species in floodplain forests investigated on three islands in the Brazilian Amazon.

According to the composition of the species recorded on the islands, 23 were exclusively observed in Costa Santana, 18 in Santaninha, and 19 species in Tauaré. In turn, the three islands share seven species between them. The number of species shared between Costa Santana/Santaninha was equal to 11 and Costa Santana/Tauaré was equal to 9. Interestingly, Santaninha presented only one species associated with Tauaré, but shared nine of them with Costa Santana (Figure 3).

Regarding species composition, the PERMANOVA showed a significant difference in fungal species composition among the islands (F = 5.678, *p* = 0.045). With a value of *p* < 0.05, we can infer that the mean composition of fungal species varies statistically among the islands, indicating that each island has a distinct set of species, which suggests specific ecological or environmental factors may be shaping these communities differently. The total variation in species composition (R^2^ = 0.45) indicates that approximately 45% can be explained by the differences among the islands.

In contrast, the PERMDISP indicates that the dispersion of species compositions relative to their centroids (the mean or central point of species composition for each island) does not differ significantly between islands (F = 1.234, *p* = 0.561). This result suggests that although the islands have distinct mean species compositions, the variability or heterogeneity within each island (i.e., how species are distributed relative to the center of their mean composition) is similar. The dispersion of species compositions on each island is homogeneous.

**Figure 4:**
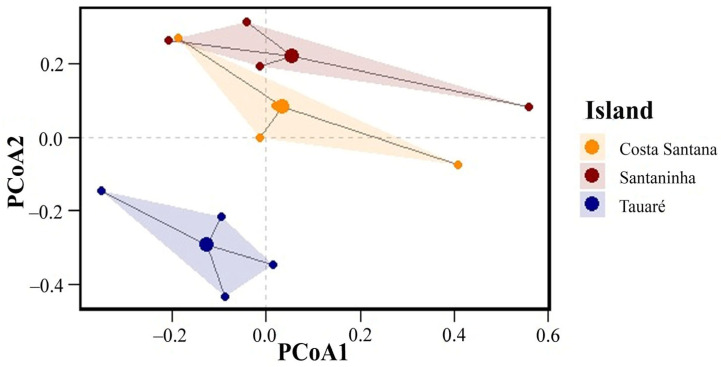
Principal coordinate analyses (PCoAs) of macrofungi composition on the three islands.

The PCoA graph (Figure 4) illustrates these results visually. The dots represent individual samples, grouped by island, with different colors. The shaded areas reflect the dispersal of species compositions on each island. The separation of the groups indicates differences in the mean composition between the islands (confirmed by PERMANOVA), while the similar size and shape of the shaded areas suggest that the internal dispersion of the species within each island is homogeneous (corroborated by PERMDISP).

Regarding species richness, the rarefaction curve, obtained by the method of the collector, revealed that the estimated species richness increased rapidly at the beginning and later stabilized around 80 species after the collection of 10 samples. Bootstrap analysis complemented this assessment by providing robust estimates of richness, averaging approximately 83.46 species. The 95% confidence interval for the bootstrap richness estimate ranged from approximately 75.04 to 90.76 species, confirming the accuracy and robustness of the estimates and indicating a reliable species richness within this range of variation.

**Figure 5:**
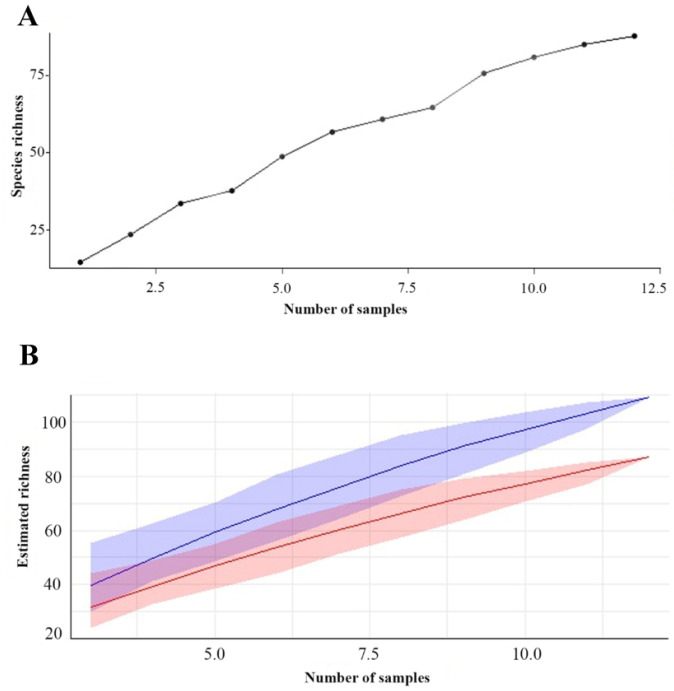
Dynamic accumulation: (**A**) Species accumulation curve in the three islands of floodplain forest. (**B**) Bootstrap chart. The red line represents the richness estimate calculated using bootstrap, while the blue line represents the observed or estimated richness directly from the data. The shaded area indicates the confidence interval.

Combined analysis of the three islands over the twelve collections reveals an accumulation curve that indicates a rapid initial increase in species richness as more samples are collected. However, as sampling progresses, the curve shows signs of stabilization, suggesting that most of the species present on the three islands were identified during the collection period. This accumulation dynamic provides valuable information about biological diversity on the islands, demonstrating that the discovery of new species tends to decline as sampling progresses (Figure 5).

### 3.2. Seasonal Diversity on the Three Islands

In relation to the differences between the dry and rainy periods, 41 species were recorded in the rainy period, 25 in the dry period, and 22 in both. In the rainy season, the highest number of species was recorded on Costa Santana with 34 species, and both Tauaré and Santaninha presented 25 species each. In the dry period, the largest number of species was recorded on Costa Santana (32 species), while Tauaré presented 22 and Santaninha 18 species.

**Figure 6:**
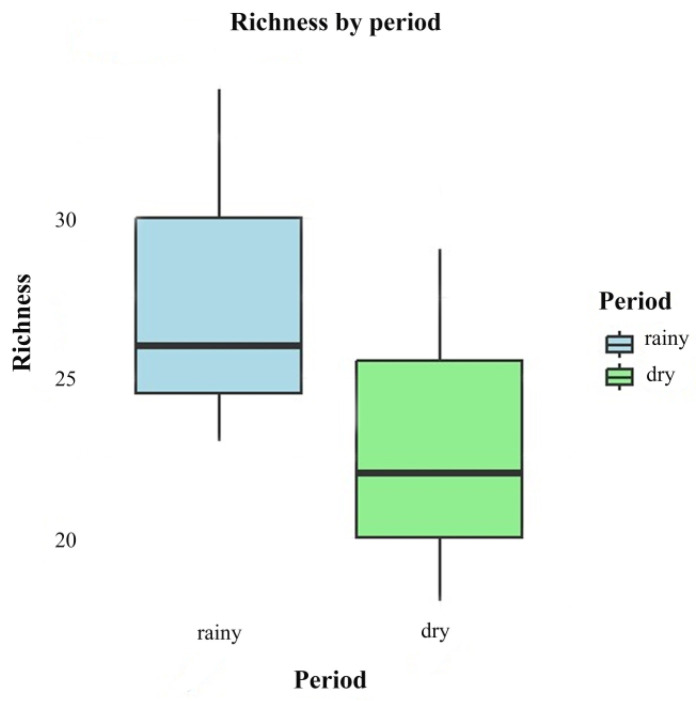
Box plots comparing the richness of fungi in different periods (rainy and dry).

There was no significant difference between the rainy season (June 2022 and 2023) and dry season (November 2022 and 2023) in relation to species richness (F = 1.032; *p* = 0.367). Although the means indicate that wealth is higher in the rainy season (mean = 27.7, standard deviation (SD) = 5.69) compared to the dry season (mean = 23.0, standard deviation (SD) = 5.57), the ANOVA indicated that this difference is not statistically significant (Figure 6).

**Figure 7:**
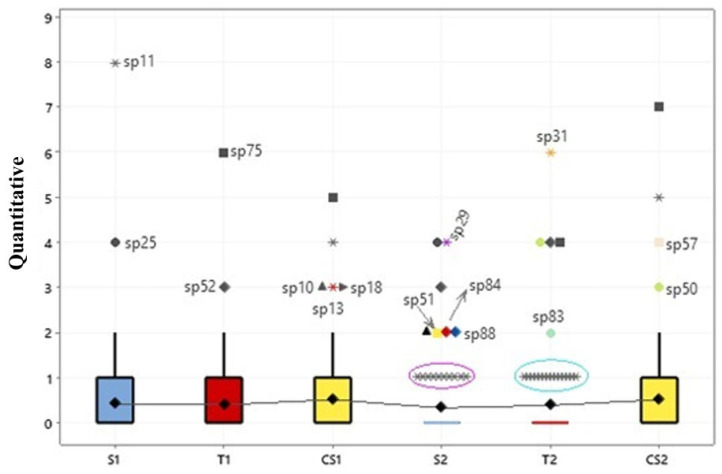
Box plots comparing the abundance of fungi on the three floodplain islands, in relation to seasonality. S = Santaninha; T = Tauaré; CS = Costa Santana; 1 = rainy season; 2 = dry season. Symbols represent the species, for example: * sp11= *Rigidoporus lineatus*.

An ANOVA was also applied to evaluate species abundance (n = 88) in relation to seasonality and there were no significant differences in the quantity of specimens of macrofungi on the islands (*p* < 0.001) (Figure 7). Nonetheless, the abundance is noticeable within each group reported via the outliers.

In Figure 7, the outliers detected in the model demonstrated the most abundant groups of macrofungi in the different seasons. In the rainy season, on Santaninha island (S1), there was the highest occurrence of the species *Trametes elegans* (sp. 25) with eight specimens and *Rigidoporus lineatus* (sp. 11) with four specimens. On the island of Tauaré (T1), the species *Earliella scabrosa* (sp. 75) and *Lentinus berteroi* (sp. 52) were the most abundant, with seven and three specimens, respectively. Finally, on the island of Costa Santana (CS1), in addition to *E. scabrosa* (five specimens) and *T. elegans* (four specimens), already recorded in S1 and T1, *Trametes maxima* (sp. 10), *Tinctoporellus isabellinus* (sp. 13), and *R. ulmarius* (sp. 18) were also the most common.

In contrast, in the dry season, the variability of these macrofungi was intensified. In Santaninha (S2), there was an increase in *Polyporus guianensis* (sp.29), *L. berteroi*, *L. concavus* (sp. 51), *T. maxima*, *Cymatoderma caperatum* (sp. 84), and *Fomes fasciatus* (sp. 88). On the island of Tauaré (T2), the most predominant macrofungi were the species *Podoscypha aculeata* (sp. 31), *L. crinitus* (sp. 50), *L. berteroi*, *E. scabrosa*, and *Lentinula raphanica* (sp. 83). On Costa Santana (CS2), the most frequent were *E. scabrosa*, *T. elegans*, *Cerrena hydnoides* (sp. 50), and *L. crinitus*.

The results of the Tukey and Fisher tests indicate a homogeneous distribution of these organisms, with certain highlights. This implies that the environmental conditions and resources available to macrofungi are similar among the islands and this reflects a balanced diversity.

## 4. Discussion

### 4.1. Composition and Richness Among the Islands

Geographical and edaphic attributes may explain the difference found in the diversity indices of the islands. The soil of Costa Santana presents high fertility in all its layers, which is fundamental for the development of plant species [41]. It has been a managed area for more than 50 years and is formed by native palms and tree species such as rubber trees, açaí palms, cocoa, and andiroba (*Carapa guianensis* Aubl.) trees and was the only one that presented the samaúma species [*Ceiba pentandra* (L.) Gaertn.—Malvaceae], a plant that is native to the Amazon. On the other hand, unlike Costa Santana, there are streams along the plantations of the Tauaré and Santaninha, characterizing a typically alluvial forest. In addition to the action of the water in the high-water period, it probably ends up selecting the plant and fungal species most adapted to the seasonal variations of the ecosystem. In fact, the specialization assumed by organisms along the floodplains causes the richness and diversity to be lower compared to the dry-land ecosystem [11,42].

In fact, the value related to species richness of macrofungi in the floodplain islands of this study was lower compared to that presented in regions of dry land in the Brazilian Amazon. Collections carried out in the Caxiuanã National Forest, located in the northwest of the state of Pará, showed indices of up to 130 species of Agaricomycetes against 88 on the floodplain islands [43,44]. Significant numbers of species were also found in Serra do Navio (100), and in Amapá National Forest (97), in the state of Amapá [45,46], as well as in a fragment of dense ombrophilous forest in the surroundings of HPP Silvio Braga, located in western Pará (91 species) [47]. However, a standardized comparative study is needed to confirm this hypothesis.

López-Quintero et al. [48] analyzed the diversity of macrofungi in two areas of the Colombian Amazon, one area being terra-firme and the other floodplains. The exclusive richness in each area was similar (n = 128), but the abundance was higher in the floodplain areas (804 sporocarps) than on dry land (741 sporocarps). The authors attributed this result to the nutrient-rich alluvial soils of the floodplains, which are due to the regular deposition of detritus in floodplain areas, and nutrients and organic matter during high/rising water, which occur on average twice a year. The authors stated that, despite the significant number of species, the rarefaction curve did not reach the asymptote, i.e., it did not saturate. This is different from the results found in this study, in which the accumulation curve stabilized from 80 species onwards.

Even if decomposing organisms are favored by organic matter and woody residues brought by river waters, it is necessary to check the richness in an area of floodplain primary forest for a more accurate assessment. Amazonian studies involving fungi associated with the floodplain ecosystem in the Brazilian Amazon, in addition to conidial microfungi [49], are scarce, especially when they report the richness and diversity of macrofungi. The data collected in this study are pioneering and fundamental for the increase in knowledge and understanding of the diversity of Agaricomycetes in floodplain forest areas, where the lack of studies involving the taxonomy of macroscopic fungi does not reflect the ecological importance of this ecosystem.

### 4.2. Seasonal Diversity

Since the field trips took place in two different periods of the year, climatological aspects probably interfered with the numbers of specimens observed in the floodplain ecosystem. In June 2022, 70 specimens of macrofungi of the class Agaricomycetes were collected, a value 25% higher when compared to November of the same year, which totaled 56. Traditionally, June presents a higher index of precipitation and humidity in the northern region in contrast to the month of November (174.4 mm/78.9%) [26]. Although there are differences in absolute numbers, statistically, this difference was not significant, as demonstrated in the ANOVA. This is different from the findings of Putra et al. [50], who evaluated the seasonal distribution of macrofungi in three forest communities in Indonesia and found significant differences. For example, of the 130 species recorded by the authors, 81 occurred in the rainy season, 25 in the dry season, and 22 in both periods. In this study, the species that were common in both periods were mainly from the Polyporaceae family (*E. scabrosa*, *L. berteroi*, *T. elegans*). Such species have a leathery and resistant basidiome and are often observed on decaying wood in open areas and/or those with intense sunlight.

Despite this, factors unrelated to climate have the potential to alter the richness and diversity of organisms in a given area. In June 2023, there was a decrease of approximately 49% in the number of specimens compared to the same period of the previous year, with 47 individuals collected. The Tauaré islands suffered the impact of the felling of its native vegetation and removal of woody material in the clearing of the land, thus reducing the supply of substrate for the growth of macrofungi. Combined with abiotic aspects, the availability of organic matter is in a relationship that is directly proportional to the number of fungi observed in the ecosystem. In other words, the greater the quantity and quality of the substrate, the greater the quantity and diversity of the fungal community [51].

Rustøen et al. [52] investigated the affinity of wood decomposing fungi in relation to the substrate of angiosperms and conifers in the United Kingdom and the effects of climate on a 40-year time scale to ascertain whether there would be compositional changes related to temporal changes and identified that the composition of wood fungi was mainly structured by the properties of the substrate, and that climatic effects were the least significant.

Although the values obtained in the diversity and richness analyses were practically the same for the three islands of Mocajuba, Costa Santana showed itself to have the highest indices, followed by Santaninha and Tauaré.

### 4.3. Future Research Directions

Floodplain forests are huge, and seasonal flood regimes, those that reach the maximum level in the wettest months, such as those that occur in the Tocantins River, limit research in these areas [53]. Although the research was not carried out in a primary floodplain area, it was carried out in an agroforestry system, which is an economic model of sustainability for preserving tree species [54]. Such preserved tree species provide ecological conditions for the maintenance of macrofungal species and, since they play a fundamental role in nutrient cycling, it is important to understand and identify diversity for the conservation and management of species for ecosystem health.

Li et al. [55] compared the diversity of macrofungi in native forests and managed plantation areas in China and noticed that the diversity was significantly higher in native forests. However, the composition of the fungal community was different between the areas. The authors point out that although diversity was the highest in native forests, the two management systems harbor distinct groups of macrofungi, suggesting that the two management methods together could provide a complementary range of macrofungi habitats.

Nonetheless, it is still necessary to compare areas of primary forests in order to discover the real distribution patterns of the species. This is a challenge in floodplain areas since they are forests that suffer from many types of anthropogenic pressures, by logging, livestock rearing and agriculture involving plants of economic interest, both for human survival and for commercialization [56]. In addition, few areas are under environmental protection, which makes it difficult to separate the effects of the impacts on populations of species.

## 5. Conclusions

Amazonian floodplain forests are rich and unique ecosystems, but they remain poorly explored regarding macrofungal diversity. Costa Santana island exhibited the highest diversity, likely associated with edaphic factors. In this study, significant differences in species composition were observed among the islands. However, no significant differences were found in the richness and abundance of the macrofungal species in relation to seasonality. Nevertheless, further investigation is needed to understand the true distribution patterns of species in the Amazon, a region severely threatened by loss of biodiversity.

## Figures and Tables

**Table 1:** Description of researched areas.

Islands/ Coordinates	Cocoa Plantation Age	Soil	Predominant Vegetation	Distance between Islands
Tauaré (02°35′56.3″ S, 49°30′46.7″ O)	15 years	Silty sand	*Hevea brasiliensis, Virola surinamensis, Pterocarpus santalinoides, Theobroma cacao, Euterpe oleracea, Carapa guianensis, Hydrochorea corymbosa, Genipa americana, Campsiandra laurifólia e Vatairea guianensis.*	8.76 km (Tauaré to Santaninha)
Santaninha (02°31′13″ S, 49°31′52″ O)	50 years	Silty sand	*Euterpe oleracea, Hevea brasiliensis, Virola surinamensis, Carapa guianensis, Pterocarpus santalinoides, Cecropia distachya, Crudia oblanga, Genipa americana, Pseudobombax munguba e Mauritia flexuosa.*	6.46 km (Santaninha to Costa Santana)
Costa Santana (02°34′37.2″ S, 49°32′20.1″ O)	50 years	Silty clay	*Theobroma cacao, Euterpe oleracea, Hevea brasiliensis, Virola surinamensis, Carapa guianensis, Pterocarpus santalinoides, Hydrochorea corymbosa, Genipa americana, Campsiandra laurifólia e Pachira aquática*	3.73 km (Costa Santana to Tauaré)

**Table 2:** List of species collected in Mocajuba. Quantity of specimens and the relative abundance (%) based on the number of specimens. Relative frequency [%] classified as follows: R = rare, O = occasional, F = frequent, and A = abundant.

Order/Family/Species	Sampled Islands			Frequency
**Agaricales** Underw	**Santaninha**	**Tauaré**	**Costa Santana**	
**Omphalotaceae** Bresinsky	**Specimens and Relative Abundance**			
* Lentinula raphanica* (Murrill) Mata and R.H. Petersen	-	2 (2.99)	2 (2.22)	O
**Pleurotaceae** Kühner				
* Pleurotus djamor* (Rumph. ex Fr.) Boedijn	-	-	1 (1.11)	R
**Schizophyllaceae** Quél.				
* Schizophyllum commune* Fr.	-	1 (1.49)	-	R
**Hymenochaetales** Oberw.				
**Hirschioporaceae** Y.C. Dai, Yuan Yuan, and Meng Zhou				
* Nigrohirschioporus sector* (Ehrenb.) Y. C. Dai, Yuan Yuan, and Meng Zhou	-	1 (1.49)	-	R
**Hymenochaetaceae** Donk				
* Fomitiporia murrillii* Alves-Silva, R.M. Silveira, and Drechsler-Santos	1 (1.52)	-	-	R
* Fomitiporia conyana* Alves-Silva and Drechsler-Santos	1 (1.52)	-	-	R
* Fomitiporia subtilissima* Alves-Silva, Reck, and Drechsler-Santos	-	1 (1.49)	-	R
* Fulvifomes kawakamii* (M.J. Larsen, Lombard, and Hodges) T. Wagner and M. Fisch.	1 (1.52)	-	-	R
* Fuscoporia licnoides* (Mont.) Oliveira-Filho and Gibertoni	-	1 (1.49)	1 (1.11)	R
* Fuscoporia scruposa* (Fr.) Gibertoni and Oliveira-Filho	1 (1.52)	-	-	R
* Hymenochaete* sp.	1 (1.52)	-	-	R
* Tropicoporus extensus* (Lév.) Y.C. Dai and F. Wu	1 (1.52)	-	-	R
**Schizoporaceae** Jülich				
* Xylodon flaviporus* (Berk. and M.A. Curtis ex Cooke) Riebesehl and Langer	-	1 (1.49)	-	R
**Polyporales** Gäum.				
**Cerrenaceae** Miettinen, Justo, and Hibbett				
* Cerrena caperata* (Berk.) Zmitr.	-	-	1 (1.11)	R
* Cerrena hydnoides* (Sw.) Zmitr.	-	1 (1.49)	4 (4.44)	O
**Fomitopsidaceae** Jülich				
* Antrodia albida* (Fr.) Donk	-	-	1 (1.11)	R
* Fomitopsis nivosa* (Berk.) Gilb. and Ryvarden	-	2 (2.99)	-	R
* Fomitopsis roseoalba* A.M.S. Soares, Ryvarden, and Gibertoni	-	-	2 (2.22)	R
* Fomitopsis* sp.	-	1 (1.49)	-	R
**Ganodermataceae** Donk				
* Amauroderma exile* (Berk.) Torrend	-	-	2 (2.22)	R
* Ganoderma australe* (Fr.) Pat.	-	1 (1.49)	1 (1.11)	R
* Ganoderma stipitatum* (Murrill) Murrill	-	-	1 (1.11)	R
**Irpicaceae** Spirin and Zmitr.				
* Ceriporia* sp.	-	-	1 (1.11)	R
**Laetiporaceae** Jülich				
* Berkcurtia persicina* (Berk. and M.A. Curtis) Robledo and Campi	-	1 (1.49)	-	R
**Meripilaceae** Jülich				
* Rigidoporus crocatus* (Pat.) Ryvarden	-	1 (1.49)	1 (1.11)	R
* Rigidoporus lineatus* (Pers.) Ryvarden	8 (12.1)	-	4 (4.44)	F
* Rigidoporus mariae* Gibertoni, Gomes-Silva and Ryvarden	1(1.52)	-	-	R
* Rigidoporus microporus* (Sw.) Overeem	-	2 (2.99)	1 (1.11)	R
* Rigidoporus ulmarius* (Sowerby) Imazeki	-	-	3 (3.33)	R
* Rigidoporus undatus* (Pers.) Donk	1 (1.52)	-	1 (1.11)	R
* Rigidoporus vinctus* (Berk.) Ryvarden	2 (3.03)	1 (1.49)	1 (1.11)	O
* Rigidoporus* sp. 1	-	-	1 (1.11)	R
* Rigidoporus* sp. 2	-	-	1 (1.11)	R
* Rigidoporus* sp. 3	-	-	1 (1.11)	R
* Rigidoporus* sp. 4	-	-	1 (1.11)	R
* Rigidoporus* sp. 5	-	-	1 (1.11)	R
**Panaceae** Miettinen, Justo, and Hibbett				
* Cymatoderma caperatum* (Berk. and Mont.) D.A. Reid	2 (3.03)	-	2 (2.22)	O
* Panus aff. fasciatus* (Berk.) Singer	-	1 (1.49)	-	R
* Panus neostrigosus* Drechsler-Santos and Wartchow	-	2 (2.99)	-	R
* Panus strigellus* (Berk.) Chardón and Toro	*-*	-	1 (1.11)	R
**Phanerochaetaceae** Jülich				
* Antrodiella angulatopora* Ryvarden	*-*	*-*	1 (1.11)	R
**Podoscyphaceae** D.A. Reid				
* Podoscypha aculeata* (Berk. and M.A. Curtis) Boidin	-	7(10.4)	1 (1.11)	O
* Podoscypha bubalina* D.A. Reid	-	1 (1.49)	1 (1.11)	R
**Polyporaceae** Fr. ex Corda				
* Cubamyces lactineus* (Berk.) Lücking	1 (1.52)	-	2 (2.22)	R
* Cubamyces menziesii* (Berk.) Lücking	-	2 (2.99)	-	R
* Cyanoporus fuligo* (Berk. and Broome) Y.C. Dai, W.L. Mao, and Yuan Yuan	-	1 (1.49)	-	R
* Earliella scabrosa* (Pers.) Gilb. and Ryvarden	1 (1.52)	10 (14.9)	12 (13.3)	A
* Fabisporus sanguineus* (L.) Zmitr	-	-	1 (1.11)	R
* Favolus trigonus* Lloyd	1 (1.52)	-	1 (1.11)	R
* Favolus tenuiculus* P. Beauv.	1 (1.52)	-	1 (1.11)	R
* Favolus pseudoprinceps* (Murrill) Sacc. and Trotter	-	1 (1.49)	-	R
* Fomes fasciatus* (Sw.) Cooke	2 (3.03)	-	-	R
* Foraminispora rugosa* (Berk.) Costa-Rez., Drechsler-Santos, and Robledo	-	1 (1.49)	-	R
* Grammothele lineata* Berk. and M.A. Curtis	1 (1.52)	-	-	R
* Lentinus atrobrunneus* Pegler	1 (1.52)	-	-	R
* Lentinus berteroi* (Fr.) Fr.	4 (6.06)	7 (10.4)	1 (1.11)	F
* Lentinus concavus* (Berk.) Corner	2 (3.03)	-	-	R
* Lentinus crinitus* (L.) Fr.	2 (3.03)	4 (5.97)	3 (3.33)	O
* Lentinus nigro-osseus* Pilát	1 (1.52)	-	1 (1.11)	R
* Lentinus scleropus* (Pers.) Fr.	1 (1.52)	-	-	R
* Lentinus* sp.1	-	1 (1.49)	-	R
* Lentinus* sp.2	1 (1.52)	-	-	R
* Lentinus swartzii* Berk.	1 (1.52)	1 (1.49)	-	R
* Lentinus tricholoma* (Mont.) Zmitr.	2 (3.03)	1 (1.49)	1 (1.11)	O
* Megasporia* sp.	1 (1.52)	-	-	R
* Megasporia cavernulosa* (Berk.) C.R.S. Lira and T.B. Gibertoni	-	1 (1.49)	-	R
* Microporellus dealbatus* (Berk. and M.A. Curtis) Murrill	-	1 (1.49)	-	R
* Perenniporia martia* (Berk.) Ryvarden	-	-	1 (1.11)	R
* Perenniporia minutopora* Ryvarden and Decock	-	-	1 (1.11)	R
* Polyporus guianensis* Mont.	4 (6.06)	-	1 (1.11)	O
* Polyporus leprieurii* Mont.	1 (1.52)	-	-	R
* Porogramme subargentea* (Speg.) Y.C. Dai, W.L. Mao, and Yuan Yuan	1 (1.52)	-	1 (1.11)	R
* Tinctoporellus isabellinus* Ryvarden and Iturriaga	1 (1.52)	-	3 (3.33)	O
* Trametes cotonea* (Pat. and Har.) Ryvarden	1 (1.52)	-	-	R
* Trametes elegans* (Spreng.) Fr.	9 (13.6)	1 (1.49)	9 (10)	F
* Trametes maxima* (Mont.) A. David and Rajchenb	2 (3.03)	1 (1.49)	5 (5.56)	O
* Trametes membranacea* (Sw.) Kreisel	-	-	1 (1.11)	R
* Trametes pavonia* (Hook.) Ryvarden	-	-	1 (1.11)	R
* Trametes supermodesta* Ryvarden and Iturr.	-	1 (1.49)	-	R
* Truncospora tephropora* (Mont.) Zmitr.	-	1 (1.49)	-	R
**Steccherinaceae** Parmasto				
* Flaviporus liebmannii* (Fr.) Ginns	-	2 (2.99)	-	R
* Junghuhnia chlamydospora* Ryvarden	-	-	1 (1.11)	R
* Nigroporus rigidus* Ryvarden	2 (3.03)	-	-	R
* Steccherinum meridionale* (Rajchenb.) Westph., Tomšovský, and Rajchenb.	-	-	1 (1.11)	R
**Russulales** Kreisel ex P.M. Kirk, P.F. Cannon, and J.C. David				
**Echinodontiaceae** Donk				
* Larssoniporia tropicalis* (Cooke) Y.C. Dai, Jia J. Chen, and B.K. Cui	1(1.52)	-	-	R
***Incertae sedis***				
* Trichaptum byssogenum* (Jungh.) Ryvarden	-	2 (2.99)	1 (1.11)	R
* Trichaptum deviatum* Ryvarden	-	-	1 (1.11)	R
* Trichaptum griseofuscum* (Mont.) Ryvarden and Iturr	1(1.52)	-	1 (1.11)	R

## Data Availability

The original contributions presented in this study are included in the article. Further inquiries can be directed to the corresponding author.
